# Algorithm based smartphone apps to assess risk of skin cancer in adults: systematic review of diagnostic accuracy studies

**DOI:** 10.1136/bmj.m127

**Published:** 2020-02-10

**Authors:** Karoline Freeman, Jacqueline Dinnes, Naomi Chuchu, Yemisi Takwoingi, Sue E Bayliss, Rubeta N Matin, Abhilash Jain, Fiona M Walter, Hywel C Williams, Jonathan J Deeks

**Affiliations:** 1 Institute of Applied Health Research, University of Birmingham, Edgbaston, Birmingham B15 2TT, UK; 2 Warwick Medical School, University of Warwick, Coventry, UK; 3 NIHR Birmingham Biomedical Research Centre, University Hospitals Birmingham NHS Foundation Trust and University of Birmingham, Birmingham, UK; 4 London School of Hygiene and Tropical Medicine, London, UK; 5 Department of Dermatology, Churchill Hospital, Oxford, UK; 6 Nuffield Department of Orthopaedics, Rheumatology and Musculoskeletal Sciences, University of Oxford, Oxford, UK; 7 Department of Plastic and Reconstructive Surgery, Imperial College Healthcare NHS Trust, St Mary’s Hospital, London, UK; 8 Department of Public Health and Primary Care, University of Cambridge, Cambridge, UK; 9 Centre of Evidence Based Dermatology, University of Nottingham, Nottingham, UK

## Abstract

**Objective:**

To examine the validity and findings of studies that examine the accuracy of algorithm based smartphone applications (“apps”) to assess risk of skin cancer in suspicious skin lesions.

**Design:**

Systematic review of diagnostic accuracy studies.

**Data sources:**

Cochrane Central Register of Controlled Trials, MEDLINE, Embase, CINAHL, CPCI, Zetoc, Science Citation Index, and online trial registers (from database inception to 10 April 2019).

**Eligibility criteria for selecting studies:**

Studies of any design that evaluated algorithm based smartphone apps to assess images of skin lesions suspicious for skin cancer. Reference standards included histological diagnosis or follow-up, and expert recommendation for further investigation or intervention. Two authors independently extracted data and assessed validity using QUADAS-2 (Quality Assessment of Diagnostic Accuracy Studies 2 tool). Estimates of sensitivity and specificity were reported for each app.

**Results:**

Nine studies that evaluated six different identifiable smartphone apps were included. Six verified results by using histology or follow-up (n=725 lesions), and three verified results by using expert recommendations (n=407 lesions). Studies were small and of poor methodological quality, with selective recruitment, high rates of unevaluable images, and differential verification. Lesion selection and image acquisition were performed by clinicians rather than smartphone users. Two CE (Conformit Europenne) marked apps are available for download. No published peer reviewed study was found evaluating the TeleSkin skinScan app. SkinVision was evaluated in three studies (n=267, 66 malignant or premalignant lesions) and achieved a sensitivity of 80% (95% confidence interval 63% to 92%) and a specificity of 78% (67% to 87%) for the detection of malignant or premalignant lesions. Accuracy of the SkinVision app verified against expert recommendations was poor (three studies).

**Conclusions:**

Current algorithm based smartphone apps cannot be relied on to detect all cases of melanoma or other skin cancers. Test performance is likely to be poorer than reported here when used in clinically relevant populations and by the intended users of the apps. The current regulatory process for awarding the CE marking for algorithm based apps does not provide adequate protection to the public.

**Systematic review registration:**

PROSPERO CRD42016033595.

## Introduction

Skin cancer is one of the most common cancers in the world, and the incidence is increasing.^1^ In 2003, the World Health Organization estimated that between two and three million skin cancers occur globally each year, 80% of which are basal cell carcinoma, 16% cutaneous squamous cell carcinoma, and 4% melanoma (around 130 000 cancers).^2^ By 2018, estimates had risen to 287 723 new melanomas worldwide.^1^ Despite its lower incidence, the potential for melanoma to metastasise to other parts of the body means that it is responsible for up to 75% of skin cancer deaths.^3^ Five year survival can be as high as 91-95% for melanoma if it is identified early,^4^ which makes early detection and treatment key to improving survival. Cutaneous squamous cell carcinoma has a lower risk of metastatic spread.^5^ ^6^ Cutaneous squamous cell carcinoma and basal cell carcinoma are locally invasive with better outcomes if treated at an early stage. Several diagnostic technologies are available to help general practitioners and dermatologists accurately identify melanomas by minimising delays in diagnosis.^7^ ^8^ The success of these technologies is reliant on people with new or changing skin lesions seeking early advice from medical professionals. Effective interventions that guide people to seek appropriate medical assessment are required.

Skin cancer smartphone applications (“apps”) provide a technological approach to assist people with suspicious lesions to decide whether they should seek further medical attention. With modern smartphones possessing the capability to capture high quality images, a wealth of “skin” apps have been developed with a range of uses.^9^ These skin apps can provide an information resource, assist in skin self examination, monitor skin conditions, and provide advice or guidance on whether to seek medical attention.^10^ ^11^ Between 2014 and 2017, 235 new dermatology smartphone apps were identified.^12^

Some skin cancer apps operate by forwarding images from the smartphone camera to an experienced professional for review, which is essentially image based teledermatology diagnosis. However, of increasing interest are smartphone apps that use inbuilt algorithms (or “artificial intelligence”) that catalogue and classify images of lesions into high or low risk for skin cancer (usually melanoma). These apps return an immediate risk assessment and subsequent recommendation to the user. Apps with inbuilt algorithms that make a medical claim are now classified as medical devices that require regulatory approval.^13^ ^14^ These apps could be harmful if recommendations are erroneous, particularly if false reassurance leads to delays in people obtaining medical assessment. CE (Conformit Europenne) marking has been applied to allow distribution of two algorithm based apps in Europe,^15^ ^16^ one of which is also available in Australia and New Zealand.^16^ However, no apps currently have United States Food and Drug Administration (FDA) approval to allow their distribution in the US and Canada. Further, the American Federal Trade Commission has fined the marketers of two apps (MelApp^17^ and Mole Detective^18^) for “deceptively claiming the apps accurately analysed melanoma risk.”

These differences in regulatory approval and false evidence claims raise questions about the extent and validity of the evidence base that supports apps with inbuilt algorithms. A previous systematic review with a search date of December 2016 examined the accuracy of mobile health apps for multiple conditions. This review identified six studies that reported on the diagnosis of melanoma, but accuracy seems to have been overestimated because it included findings from both development and validation studies.^9^ In our review, we aim to report on the scope, findings, and validity of the evidence in studies that examine the accuracy of all apps that use inbuilt algorithms to identify skin cancer in users of smartphones.^19^

## Methods

This review extends and updates our systematic review of smartphone apps^19^ (which was limited to diagnosis of melanoma). We conducted our review according to methods detailed in the Cochrane Handbook for Systematic Reviews of Diagnostic Test Accuracy^20^ and report our findings according to the Preferred Reporting Items for Systematic Reviews and Meta-Analyses (PRISMA) extension for diagnostic test accuracy studies statement recommendations.^21^

### Data sources

We conducted literature searches for our original Cochrane review from inception of the databases to August 2016.^19^ For this review, we carried out an updated search for studies published between August 2016 and 10 April 2019. The databases searched were the Cochrane Central Register of Controlled Trials, MEDLINE, Embase, CINAHL, CPCI, Zetoc, Science Citation Index, US National Institutes of Health Ongoing Trials Register, NIHR Clinical Research Network Portfolio Database, and the World Health Organization International Clinical Trials Registry Platform. Online supplementary appendix 1 presents the full search strategies. We did not apply any language restrictions. The reference lists of systematic reviews and included study reports were screened for additional relevant studies.

### Study selection

Two reviewers independently screened the titles and abstracts of all retrieved records, and subsequently all full text publications. Discrepancies were resolved by consensus or discussion with a third reviewer. Studies of any design that evaluated algorithm or “artificial intelligence” based smartphone apps that used photographs (that is, macroscopic images) of potentially malignant skin lesions were eligible for inclusion if they provided a cross tabulation of skin cancer risk against a reference standard diagnosis. Reference standards were either histological diagnosis with or without follow-up of presumed benign lesions (estimating diagnostic accuracy), or expert recommendation for further investigation or intervention (eg, excision, biopsy, or expert assessment). Studies that used a smartphone magnifier attachment were excluded on the basis that such attachments are relatively uncommon among smartphone users, and are more often used in high risk populations for lesion monitoring. Studies developing new apps were excluded unless a separate independent “test set” of images was used to evaluate the new approach. Conference abstracts were excluded unless associated full texts could be identified. Online supplementary appendix 2 presents a list of excluded studies with reasons for exclusion. We contacted the authors of eligible studies when they presented insufficient data to allow for the construction of 2×2 contingency tables or for supporting information not reported in the publication.

### Data collection, quality assessment, and analysis

Two authors independently extracted data by using a prespecified data extraction form and assessed study quality. For diagnostic accuracy, each study would ideally have prospectively recruited a representative sample of patients who used the app on their own smartphone device to evaluate lesions of concern. Verification of results (blinded to the apps’ findings), to determine whether each lesion evaluated was skin cancer or not, would have been conducted by****using histological assessment (if excised) or follow-up (if not excised). For verification with expert recommendations, all lesions assessed by the app would be reassessed in person by an expert dermatologist. Data would be reported for all lesions, including those for which the app failed to provide an assessment. These aspects of study quality were assessed using the Quality Assessment of Diagnostic Accuracy Studies 2 tool (QUADAS-2^22^; online supplementary appendix 3). Any disagreements were resolved by consensus.

We plotted estimates of sensitivity and specificity from each study on coupled forest plots for each variation of each app. The app recommendations associated with the risk of melanoma for the different apps were tabulated (table 1). When apps reported three risk categories (high, moderate, low risk), we used the recommendations provided by each app to decide whether moderate risk results from the app should be combined with low or high risk results for the estimation of test accuracy. In cases of ambiguity, both options were pursued. Because of scarcity of data and poor quality of studies, we did not perform a meta-analysis. Forest plots were produced using RevMan 5.3 (Nordic Cochrane Centre). We present data on a per lesion basis.

**Table 1:** Summary of recommendations for low, moderate, and high risk lesions by named algorithm based apps identified by this review

App	Platform; app availability	Low risk	Moderate risk	High risk	Comparison
Currently available apps					
skinScan*	iOS; Europe (CE marked), Australia, and New Zealand	“Typical”	—	“Atypical”	—
SkinVision† with or without questionnaire	iOS, Android; Europe (CE marked)	“Not much to worry about”; monitor for any changes	“Some chaotic growth”; consult a doctor	“Abnormal growth”; consult a doctor asap	H *v* M/L H/M *v* L
Apps with uncertain availability (urls not accessible)					
Dr Mole‡§	Android/Amazon; app last updated 2 August 201*¶	No specific action recommended	Consult specialist	Consult specialist immediately	H/M *v* L
SpotMole‡	Android; app last updated 30 March 2016**	Okay; see a doctor if still concerned	—	“Problematic”; consult doctor	H *v* L
Apps withdrawn from market					
MelApp‡	iOS, Android	Low	Medium	High	H *v* M/L H/M *v* L
Mole Detective‡	iOS, Android	Monitor; no consultation needed	“Symptoms of melanoma”; monitor and schedule annual dermatology appointment	“Several symptoms of melanoma”; consult dermatologist	H *v* M/L H/M *v* L

CE=Conformit Europenne; H=high risk; L=low risk; M=moderate risk.

* This is the TeleSkin skinScan app and not the SkinScan app evaluated by Chadwick and colleagues.^23^ No published peer reviewed study was found evaluating the TeleSkin skinScan app. TeleSkin skinScan video published 18 January 2013 (https://youtu.be/xyOdAJnIPqA).

† SkinVision YouTube video published 17 August 2017 (https://youtu.be/DqrGkJj1eEE).

‡ Risk recommendations as reported by Chadwick and colleagues^23^ (no specific actions were identified for MelApp).

§ Ngoo and colleagues^24^ report results for Dr Mole as percentage of bar filled (continuous risk) and selects cut-off percentage of 72.5% (sensitivity=specificity).

¶ https://apkpure.com/doctor-mole-skin-cancer-app/com.revsoft.doctormole ; https://www.amazon.com/Doctor-Mole/dp/B007P8GA36.

** https://play.google.com/store/apps/details?id=com.spotmole&hl=en_GB.

### Patient and public involvement

The protocol for the review^19^ ^25^ was developed and written with input from two coauthors with lived experience of skin cancer to ensure that due consideration was given to the patient and public perspective.

## Results

### Study selection

The search identified 418 unique records of which 64 were selected for full text assessment along with 16 studies identified for the original review (see online supplementary fig 1 for full PRISMA flow diagram). We contacted corresponding authors for further information on three studies. Responses were received from two authors, and one provided additional relevant information. We excluded more than a third of studies (30/80, 37.5%) on the basis of the index test. Reasons for exclusion were because studies did not evaluate smartphones or smartphone apps (n=18); they were development studies without independent validation (n=6); they used magnifying attachments to the phone camera (n=3); they operated on a store and forward teledermatology basis (n=2); or they were used for lesion monitoring (n=1). Two studies^26^ ^27^ duplicated data included in other studies.^28^ ^29^ The supplementary figure and online supplementary appendix 2 document the other reasons for exclusion.

### Characteristics of included studies

Nine studies (9/80, 11.3%) met eligibility criteria.^23^ ^24^ ^28^ ^29^ ^30^ ^31^ ^32^ ^33^ ^34^ Six studies (including 725 skin lesions) evaluated the diagnostic accuracy of smartphone apps for risk stratification of suspicious skin lesions by comparing app risk gradings with a histopathological reference standard diagnosis (some incorporated clinical expert face to face diagnosis for some lesions).^23^ ^28^ ^31^ ^32^ ^33^ ^34^ Five of the six studies aimed to detect melanoma only, and one^33^ aimed to differentiate between malignant (including melanoma, basal cell carcinoma, and squamous cell carcinoma) or premalignant lesions and benign lesions. Three studies (with 407 lesions) verified the smartphone app recommendations against a reference standard of expert recommendations for further investigation or intervention (identification of a lesion as malignant or premalignant,^30^ histology required or not,^29^ or a face to face consultation required or not^24^).

Studies evaluated six different named apps. Table 1 summarises these apps according to current availability. The app named SkinScan in Chadwick 2014^23^ is a predecessor of SkinVision and not the CE marked TeleSkin skinScan app. Only the TeleSkin skinScan and SkinVision apps are currently available; MelApp and Mole Detective were withdrawn from the market after American Federal Trade Commission investigations^17^ ^18^; and Dr Mole and Spotmole appear to be no longer available. Two studies assessed one^31^ and three^34^ apps without disclosing their names.

Table 2 and table 3 summarise the characteristics of the included studies. Sample sizes ranged from 15^23^ to 199 lesions,^30^ with up to 45%^30^ of lesion images reported as unevaluable. After exclusions, the mean number of included lesions was 91 (median 108). Three studies reported between five^30^ and 10^23^ ^24^ attempts to obtain an adequate image for each lesion, and one study^28^ analysed a minimum of three images for each lesion. Two studies included any type of skin lesion,^30^ ^33^ and all other studies restricted inclusion to pigmented or melanocytic lesions only.

**Table 2:** Characteristics of studies that reported diagnostic accuracy of smartphone apps verified by histology with or without follow-up

Study, country	Apps	No of patients, lesions	Inclusion criteria	Exclusion criteria	Data collection	Choice of lesions, image acquisition	Reference standard, target condition	No of exclusions (%)	No of cancers/total analysed, % (final diagnoses)
Chadwick (2014),^23^ Australia	SkinScan, Mel App, Mole Detective, Spot Mole Plus, Dr Mole Premium	NR, 15	Images of melanocytic lesions excised with histopathological diagnosis	“Unable to analyse” lesions after 10 attempts	Retrospective (prospective interpretation)	Clinician, clinician	Histopathology (no FU), melanoma *v* benign naevus	Unevaluable images excluded a priori NR	5/15, 33.3% (5 MM or MiS, 10 BN)
Dorairaj (2017),^31^ Ireland	App (not named*)	32, 32	Patients referred for excision of pigmented lesions	NR	Prospective	NR, clinician	Histopathology (no FU), melanoma *v* dysplastic naevus or benign	Unevaluable images 6 (19%)	9/26, 35% (9 MM or MiS, benign diagnoses NR)
Maier (2015),^28^ Germany	SkinVision (original version)	NR, 195‡	Patients with melanocytic skin lesions seen routinely for skin cancer screening at dermatology department	Poor quality index test image; other elements in the image, eg, hair, images containing more than one lesion, incomplete imaged lesions, non-melanocytic lesions, two point differences cases, tie cases	Prospective	Clinician, clinician	Histopathology (no FU), melanoma *v* not melanoma	Unevaluable images 20 (10%); “tie” cases 18 (9%); two point differences 13 (7%)	26/144, 18.1% (26 MM or MiS, 34 DN, 84 BN)
Robson (2012),^32^ United Kingdom	MelApp	31, 35	Patients with pigmented skin lesions referred from GPs to urgent cancer clinic	NR	Prospective	NR, clinician	Histopathology (49%) or clinical assessment (no FU), melanoma *v* not melanoma	Unevaluable images 14 (40%)	2/21, 9.5% (histology: 2 MM, 4 BN, 2 DN, 1 blue naevus, 2 SK; clinically benign 10)
Thissen (2017),^33^ Netherlands	SkinVision (original and rev±qu)†	256, 341§	Patients with pigmented or non-pigmented skin lesions seen routinely at the dermatology department	NR	Prospective	Clinician, clinician	Histopathology (38%) or clinical assessment (no FU), malignant or premalignant *v* benign	None excluded owing to quality to mimic real world use	35/108, 32.4% (malignant or premalignant: 2 MM, 1 MiS, 16 BCC, 3 cSCC, 5 BD, 8 AK, all with histology apart from 2 BCC and 3 AK; benign: 9 SK, 12 BN, 1 DN, 7 SL, 3 LPLK, 41 other benign¶)
Wolf (2013),^34^ United States	3 Apps (not named)	NR, 188	Images of pigmented skin lesions with a clear histological diagnosis assessed by a board certified dermatopathologist	“Difficult to diagnose” lesions, lesions with equivocal diagnoses, specific lesion types, eg, SN or atypical naevi (moderate high grade), images with identifiable features	Retrospective (prospective interpretation)	Clinician, clinician	Histopathology (no FU), melanoma *v* benign lesions	Unevaluable images app 1: 6 (3%); app 2: 3 (2%); app 3: 17 (9%); plus poor quality excluded a priori	60/188, 31.9% (44 MM, 16 MiS, 94 BN, 20 SK, 8 SL, 2 hemangiona 2, 4 DF)

The Chadwick 2014 SkinScan app is a predecessor of SkinVision and not related to the CE marked TeleSkin skinScan app. AK=actinic keratosis; BCC=basal cell carcinoma; BD=Bowens disease; BN=benign naevi; cSCC=cutaneous squamous cell carcinoma; DF=dermatofibroma; DN=dysplastic naevi; FU=follow-up; GP=general practitioner; LPLK=lichen planus like keratosis; MiS=melanoma in situ; MM=malignant “invasive” melanoma; NR=not reported; SK=seborrhoeic keratosis; SL=solar lentigo; SN=Spitz naevi.

* App name not declared in study and could not be divulged by the authors (J Dorairaj, personal communication, 2019).

† SkinVision (rev±qu)=revised version of the SkinVision app, “recalibrated” to accommodate non-pigmented lesions and including the option of a questionnaire about lesion characteristics (eg, texture, colour, shape, size, and symptoms).

‡ At least three images per lesion.

§ 108/341 included as test set.

¶ 41 other benign include 10 psoriasis, 8 histiocytoma, 8 folliculitis, 3 sebaceous hyperplasia, 6 angioma senilis, 4 scars, 1 clear cell acanthoma, 1 verruca vulgaris.

**Table 3:** Characteristics of studies that reported accuracy of smartphone apps verified by expert recommendations for further investigation or intervention

Study, country	Apps	No of patients, lesions	Inclusion criteria	Exclusion criteria	Data collection	Choice of lesions, image acquisition	Reference standard, target condition	No of exclusions (%)	No of cancers/total analysed, % (final diagnoses)
Chung (2018),^30^ Netherlands	SkinVision (version NR)	125, 199	Visitors of the National Skin Cancer Day (up to 2 lesions selected for assessment by attendees)	NR	Prospective	Patient, clinician	Expert assessment (appears to be face to face), malignant or premalignant *v* benign	Unevaluable images 90 (45%)*	9/109, 8.3% (final diagnoses NR; expert diagnoses: 6 BCC, 1 BD, 1 AK, 1 angioma plus 54 BN, 7 atypical BN, 21 SK, 8 SL, 7 DF, 3 other BN)
Nabil (2017),^29^ Netherlands	SkinVision (version NR)	NR, 151	New patients referred by GP to the pigmented lesion clinic (up to 2 lesions selected for assessment by attendees)	NR	Prospective	Patient, clinician	Expert assessment in face to face consultation, histopathology warranted *v* no histopathology	No unevaluable images reported	8/151, 5.3% (final diagnoses NR; expert diagnoses obtained from author: 5 MM, 3 BCC, 3 AK, 3 DF, 17 DN, 86 BN, 3 angioma, 4 SL, 2 blue naevus, 1 SN, 1 giant comedo)
Ngoo (2018),^24^ Australia	SkinVision (version NR), SpotMole, Dr Mole	30, 57	Surgical list patients with pigmented lesions scheduled for excision, participants from naevus morphology study	Lesions on non-typical skin surfaces, poor image quality, keratinocyte lesions	Prospective	Clinician, clinician	Expert assessment of dermoscopic and clinical images, lesion warrants in-person consultation *v* benign	Poor quality images excluded a priori 4/38 (11%), plus 3 participants with ineligible lesions and 1 with no excision; unevaluable images: SkinVision iOS 8 (14%), SkinVision Android 10 (18%)†	42/57, 73.7% (expert diagnoses NR; histology reported 1 MiS)

AK=actinic keratosis; BCC=basal cell carcinoma; BD=Bowens disease; BN=benign naevi; DF=dermatofibroma; DN=dysplastic naevi; GP=general practitioner; MiS=melanoma in situ; MM=malignant “invasive” melanoma; NR=not reported; SK=seborrhoeic keratosis; SL=solar lentigo; SN=Spitz naevi.

* Up to five attempts for each lesion.

† Up to 10 attempts for each lesion.

### Assessment of the validity and applicability of the evidence using QUADAS-2

**Fig 1:**
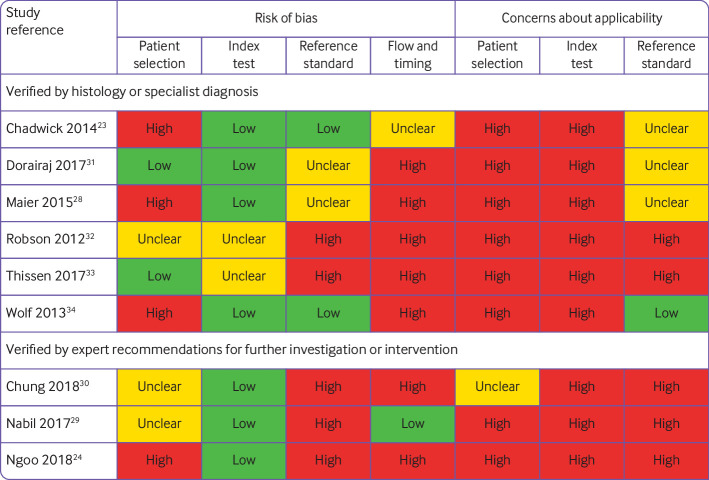
Overview of risk of bias and applicability concerns of included studies

Only four studies^28^ ^31^ ^33^ ^34^ recruited a consecutive sample of study participants or lesions. The lesion selection process was otherwise unclear^29^ ^30^ ^32^ or convenience sampling was used.^23^ ^24^ One prospective study recruited patients from the general population who attended a national skin cancer day held at three university medical centres^30^; one recruited patients who attended follow-up screening^28^; and seven recruited only patients selected for excision of suspicious lesions or assessment of skin problems by dermatologists.^23^ ^24^ ^29^ ^31^ ^32^ ^33^ ^34^ Only two studies included skin lesions as selected by study participants (up to two for each participant)^29^ ^30^; two did not report lesion selection^31^ ^32^; and in five,^23^ ^24^ ^28^ ^33^ ^34^ the clinician performed lesion selection. Only two studies were rated to be at low risk of bias for patient selection, and in eight of the nine studies, the selection of skin lesions for assessment did not reflect the lesions that would be assessed in the population who might use the smartphone apps (fig 1).

We had high (eight of nine) or unclear (one of nine) concerns about the application of the index test. In seven studies^24^ ^28^ ^29^ ^30^ ^31^ ^32^ ^33^ researchers, rather than study participants, used the app to photograph lesions. Two studies used previously acquired images of excised lesions obtained from dermatology databases,^23^ ^34^ which raised concerns that the results of the studies were unlikely to be representative of real life use. Image quality was likely to be higher than in the real life setting for two reasons: archived images were chosen on the basis of image quality; or a clinician or researcher prospectively acquired images by using a standard protocol under optimised conditions and using a single smartphone camera rather than by participants using their own individual devices. Studies reduced the number of non-evaluable images by attempting up to 10 image submissions for each lesion. One study considered the results of a minimum of three images for each lesion in the final risk assessment.^28^

Most diagnostic accuracy studies (n=5) aimed to differentiate between melanomas and benign lesions^23^ ^28^ ^31^ ^32^ ^34^; one study^33^ included other types of skin cancer and premalignant lesions as the target condition. The risk of bias for the reference standard was low in only two studies.^23^ ^34^ Five studies used expert diagnosis to confirm the final diagnosis for at least some lesions, with no confirmation by the preferred reference standard of histopathology or lesion follow-up.^24^ ^29^ ^30^ ^32^ ^33^ Additionally in five studies it was unclear whether the final diagnosis had been made without any knowledge of the app result.^28^ ^29^ ^31^ ^32^ ^33^

Exclusion of unevaluable images for which the app could not return a risk assessment might have systematically inflated the diagnostic performance of the tested apps in six of the nine papers.^24^ ^28^ ^30^ ^31^ ^32^ ^34^ Four studies reported exclusion criteria,^23^ ^24^ ^28^ ^34^ which included difficult to diagnose conditions; poor image quality; and unequivocal results obtained from the apps. For example, Maier and colleagues^28^ assessed a minimum of three images for each lesion. They excluded lesions with images from the same lesion falling into high and low risk categories, and tie cases, when the three images from a single lesion are categorised at different risk levels (high, medium, and low risk).

### Study synthesis: sources of variability in test performance

All but two of the identifiable apps report lesion recommendations as high, moderate, or low risk (table 1); SpotMole and skinScan do not feature a moderate risk result. We were unable to identify the action recommended for a high risk result from MelApp, and have assumed that, as for other apps, users would (be recommended to) consult a doctor. For moderate risk lesions, one app recommends lesion monitoring (Mole Detective) and two recommend consulting a doctor (SkinVision and Dr Mole), although with less urgency than implied for a high risk result.

Other sources of variability included varying definitions of the target condition (any malignant or premalignant lesion in one study,^33^ and melanoma only in five studies); different app versions (adaptations to improve the performance of apps for non-pigmented lesions and apps for different mobile phone platforms); consideration of results of a short user questionnaire; and mode of image upload (directly into the app *v* indirectly from the phone’s internal storage).

### Test performance of algorithm based skin cancer apps

**Fig 2:**
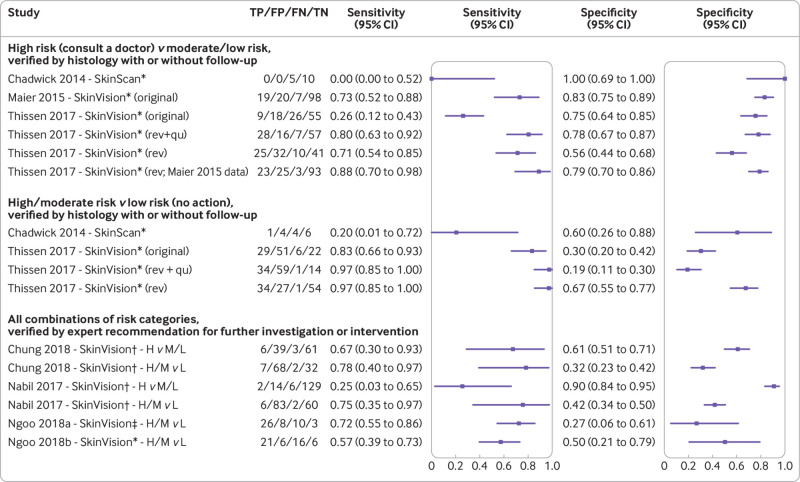
Forest plot estimates of sensitivity and specificity for studies of currently available algorithm based apps. The Chadwick 2014 SkinScan app is a predecessor of SkinVision and not related to the CE marked TeleSkin skinScan app. Data are presented when only high risk results were considered as test positive, or when high and moderate risk results were considered as test positive. Unevaluable images: Chadwick 2014: excluded a priori; Chung 2018: 90; Maier 2015: 20; Nabil 2017: not reported; Ngoo 2018a: 10; Ngoo 2018b: 8; Thissen 2017: 0. FN=number of people with a false negative result; FP=number of people with a false positive result; H=high risk; L=low risk; M=moderate risk; rev=revised (version of the app); rev+qu=revised (version of the app) plus participant responses to questions about their skin lesion; TN=number of people with a true negative result; TP=number of people with a true positive result. *iOS; †mobile platform not reported; ‡Android

Figure 2 presents the results of the apps that are currently available on a per lesion basis. No published peer reviewed study was found evaluating the TeleSkin skinScan app. The SkinVision predecessor app SkinScan was evaluated in a single study of only 15 lesions (five melanomas).^23^ Sensitivity was low regardless of whether moderate risk was combined with the low or high risk category (0% or 20% respectively), with corresponding specificities of 100% and 60% (fig 2). When only high risk results were considered as test positive, the original SkinVision app demonstrated a sensitivity of 73% (95% confidence interval 52% to 88%) in a study of pigmented lesions (n=144, 26 melanomas)^28^; however, sensitivity was only 26% (12% to 43%) when applied to pigmented and non-pigmented lesions (n=108, 35 malignant or premalignant lesions).^33^ The app only correctly picked up one of three melanomas as high risk.^33^ Corresponding specificities were 83% and 75% (fig 2). A later revision of the app to allow for non-pigmented lesions led to a 15 percentage point increase in sensitivity for the detection of melanoma when applied to the original pigmented lesion dataset (88%, 95% confidence interval 70% to 98%); however, specificity dropped by 3 percentage points (79%, 70% to 86%).^33^ Additionally, sensitivity for the detection of malignant or premalignant lesions increased by 45 percentage points when applied to the pigmented and non-pigmented lesion dataset (71%, 54% to 85%), but specificity dropped by 19 percentage points (56%, 44% to 68%).^33^ When participant responses to in-app questions about lesion characteristics and symptoms were included, sensitivity increased further to 80% (63% to 92%) and specificity to 78% (67% to 87%).^33^

When the interpretation of SkinVision was varied to consider high and moderate results from the app as test positive, sensitivity increased (between 17 and 57 percentage points) but at a considerable cost to specificity (falling by 11-45 percentage points).^33^

Three studies assessed the SkinVision app.^24^ ^29^ ^30^ The app was verified against expert recommendations for further investigation or intervention, presumably using the original version of the app.^24^ ^29^ ^30^ Agreement between the app and the expert lesion assessment was poor and variable regardless of the threshold for test positivity applied (fig 2). When only high risk results were considered as test positive, between 25% (95% confidence interval 3% to 65%)^29^ and 67% (30% to 93%)^30^ of lesions that the dermatologist considered to require further investigation were picked up by the app (with specificities of 90% and 61%, respectively). When high and moderate risk results were considered as test positive, sensitivities ranged from 57% to 78%, and specificities from 27% to 50% (fig 2).

Results from the five studies that reported data for apps with uncertain availability^23^ ^24^ or withdrawn apps^23^ ^32^ are if anything more variable. These variable results could partly be caused by smaller sample sizes, with either low sensitivities (25-50% for MelApp) or specificities (20-60% for Mole Detective, Dr Mole, and SpotMole). Online supplementary figure 2 presents the results for these studies and for the evaluations of unidentifiable apps.^31^ ^34^

### Test failure

When apps failed to return a risk assessment, images were either excluded a priori from the studies,^23^ excluded from analysis,^24^ ^28^ ^30^ ^31^ ^32^ ^34^ or were not reported.^29^ Table 2, table 3, and figure 2 report the numbers excluded for each analysis because of test failure, which ranged from 3/188 (1.6%)^34^ to 90/199 (45.2%).^30^ Only one study^33^ reported analysing all images to more closely mimic a real world setting.

## Discussion

### Main findings

In this systematic review of algorithm based smartphone apps we found nine studies that evaluated six named apps for risk stratification of skin lesions. Only two CE marked apps are known to be currently available for download in various parts of the world. Evaluations of apps with unknown availability and of those now withdrawn from the market because of “deceptive claims” were particularly small and with highly variable results. Our review shows small improvements over time in the diagnostic accuracy of one currently available app (SkinVision) and a stark lack of valid evidence for the other app (SkinScan). Identified studies of test accuracy have many weaknesses and do not provide adequate evidence to support implementation of current apps.

Despite the limitations of the evidence base, two algorithm based apps have obtained the CE marking and are currently being marketed with claims that they can “detect skin cancer at an early stage”^15^ or “track moles over time with the aim of catching melanoma at an earlier stage of the disease”.^16^ Under the EU Medical Device Directive^35^ smartphone apps are class 1 devices. Manufacturers can apply CE marking to class 1 devices as long as they have shown compliance with the “essential requirements” as outlined in the Directive,^35^ and without necessarily being subject to independent inspection by notified bodies such as the Medicines and Healthcare products Regulatory Agency in the United Kingdom. Under the new Medical Device Regulations,^36^ ^37^ which come into full force by May 2020, smartphone apps could be in higher device classes and will be subject to inspection by notified bodies. The FDA already has a stricter assessment process to evaluate mobile apps by taking a wider perspective of harm where “functionality could pose a risk to a patient’s safety if the mobile app were to not function as intended”.^13^ No skin cancer risk stratification smartphone app has received FDA approval to date.

Across the body of evidence presented, different apps recommended conflicting management advice for the same lesions.^23^ ^34^ Additionally app recommendations commonly disagreed with histopathological results or clinical assessment, with some apps unable to identify any cases of melanoma.^23^

The SkinVision app produced the highest estimates of accuracy. Therefore, in a hypothetical population of 1000 adults in which 3% have a melanoma, four of 30 melanomas would not be picked up as high risk, and more than 200 people would be given false positive results (by using a sensitivity of 88% and a specificity of 79%, as observed by Thissen and colleagues^33^). However, performance is likely to be poor because studies were small and overall of poor methodological quality, and did not evaluate the apps as they would be used in practice by the people who would use them. Selective participant recruitment, inadequate reference standards, differential verification, and high rates of unevaluable images were particular problems.

### Challenges in evaluation studies

Firstly, smartphone apps are typically targeted at the general population with a relatively low prevalence of malignant lesions and a wide range of different skin conditions. Studies failed to recruit samples representative of this population. We found studies were based on images of suspicious skin lesions that had undergone excision or biopsy, and were further selected to only include conditions identified by the apps; for example, they excluded lesions with clinical and histological features similar to melanoma,^34^ or restricted inclusion to melanocytic lesions which are more likely to be recognised by apps.^23^ ^28^ ^29^ ^31^ ^32^ Such fundamental differences in the spectrum of skin conditions compared with the general population means that poorer accuracy is likely to be observed in a real world setting.^38^ ^39^ Study results are also not applicable to people with amelanotic melanomas (accounting for 2-8% of all melanomas),^40^ or to identify other more common forms of skin cancers such as cutaneous squamous cell carcinoma.

Secondly, image quality is a major concern for smartphone apps. Smartphone cameras are much more likely to be used in suboptimal conditions by the general population, which results in variable image quality. Even under controlled conditions, studies reported difficulties in obtaining clear images of large lesions, erosive surface of ulcerated tumours, mottled skin, lesions in skin folds, tanned skin, or multiple lesions in close approximation. These problems resulted in image exclusion and potential overestimation of diagnostic performance of skin cancer apps. The analysis of less than optimal images when used by smartphone users will further affect the ability of skin cancer apps to accurately differentiate between high risk and low risk lesions.

Thirdly, the lack of clarity in smartphone app recommendations could leave concerned users uncertain as to the best course of action. Reactions to a moderate risk result will probably depend on how risk averse people are, which will most likely result in variable decisions, with a risk of people failing to present to a specialist with a potentially malignant skin lesion. Therefore, predicting the true performance of the different apps in a real world setting is impossible without further research into behavioural responses in different groups of people with a range of lesion types.

Fourthly, algorithm based apps are constantly evolving. An evaluation of an app version and insights into its performance might not be applicable to the version available to users. Studies included in this review did not specify algorithm versions used for risk assessment and we do not know whether three studies of the SkinVision app^29^ ^30^ ^33^ considered the same app version or different versions.

Finally, the potential benefit of smartphone apps lies in their availability and use by people outside the healthcare system to evaluate lesions that cause them concern. However, all studies evaluated lesions or images selected and acquired by clinicians rather than lesions judged to be of concern to people using the apps. Concern exists about the impact of false reassurances that algorithm based apps could give users with potentially malignant skin lesions, especially if they are dissuaded from seeking healthcare advice. These patients are not represented in the reported studies and thus we did not evaluate this risk. A considerable number of users will receive an inappropriate high risk result that could cause unnecessary worry and a burden on primary care and dermatology services.

Algorithms within current apps can be improved and might well reach performance levels suited for a screening role in the near future. However, manufacturers and researchers need to design studies that provide valid assessments of accuracy. A SkinVision study^41^ published after our search was conducted reported improved estimates of sensitivity for the detection of malignant or premalignant lesions for a new version of the app (95.1%, 95% confidence interval 91.9% to 97.3%) with similar specificity (78.3%, 77.3% to 79.3%). The study has used some images and data collected by real users of the app on their own phones, however the selection of malignant and benign lesions from several different sources is likely to have introduced bias. Two thirds (195/285) of the malignant or premalignant lesions originated from previous studies (including 40 melanomas from one study^28^ and eight melanomas plus 147 other malignant or premalignant lesions from another study^33^), with images taken by experts on patients referred to a clinic. Another 90 melanomas were identified from users of the app who had uploaded histology results after a high risk rating by a dermatologist and a high or moderate risk recommendation from the app (which will overestimate accuracy if more easily identifiable melanomas were included).^41^ Clinical assessment by a dermatologist of a single image submitted online with no histology, no in-person assessment, or follow-up identified the 6000 apparently benign lesions included. Therefore, it is possible that this group might include some missed melanomas.

### Strengths and weaknesses of this review

The strengths of this review are that it used a comprehensive electronic literature search with stringent systematic review methods that included independent duplicate data extraction and quality assessment of studies, attempted contact with authors, and a clear analysis structure. We included an additional two studies^29^ ^30^ that were not included in previous reviews. We also excluded between three^42^ and five^9^ studies that reported the development (without independent validation) of new apps that were included in the other two reviews because such studies are likely to overestimate accuracy.

We included studies that verified app findings by using expert recommendations for investigation or intervention, in addition to studies that used histology with or without follow-up. Studies that used histology and follow-up are more reliable because the app assessments are evaluated against the true final diagnosis of study lesions; low sensitivities or specificities reflect the apps’ inability to identify melanomas or other skin cancers as high risk. Studies that verify findings against expert recommendations can only provide an idea of the level of agreement between the algorithm’s risk assessment and a dermatologist’s clinical management decision; agreement between the two does not necessarily mean the risk assessment made was the correct one for the lesion concerned.

### Implications for practice

Despite the increasing availability of skin cancer apps, the lack of evidence and considerable limitations in the studies ultimately highlight concerns about the safety of algorithm based smartphone apps at present. The generalisability of study findings is of particular concern. Investment in algorithm based skin cancer apps is ongoing, with the company behind SkinVision announcing an investment of US$7.6m (£5.8m; €6.8m) in 2018.^43^ Subsequently, in March 2019,^44^ this app was selected to join the UK NHS Innovation Accelerator as a possible new technology to support earlier diagnosis and prevention of cancer. Therefore, it is vital that healthcare professionals are aware of the current limitations in the technologies and their evaluations. Regulators need to become alert to the potential harm that poorly performing algorithm based diagnostic or risk stratification apps create.

### Implications for research

Future studies of algorithm based smartphone apps should be based on a clinically relevant population of smartphone users who might have concerns about their risk of skin cancer or who could have concerns about a new or changing skin lesion. Lesions that are referred for further assessment and those that are not must be included. A combined reference standard of histology and clinical follow-up of benign lesions would provide more reliable and more generalisable results. Complete data that include the failure rates caused by poor image quality must be reported. Any future research study should conform to reporting guidelines, including the updated Standards for Reporting of Diagnostic Accuracy guideline,^45^ and relevant considerations from the forthcoming artificial intelligence specific extension to the CONSORT (Consolidated Standards of Reporting Trials) statement.^46^

### Conclusion

Smartphone algorithm based apps for skin cancer all include disclaimers that the results should only be used as a guide and cannot replace healthcare advice. Therefore, these apps attempt to evade any responsibility for negative outcomes experienced by users. Nevertheless, our review found poor and variable performance of algorithm based smartphone apps, which indicates that these apps have not yet shown sufficient promise to recommend their use. The current CE marking assessment processes are inadequate for protecting the public against the risks created by using smartphone diagnostic or risk stratification apps. Smartphones and dedicated skin cancer apps can have other roles; for example, assisting in skin self-examination, tracking the evolution of suspicious lesions in people more at risk of developing skin cancer,^47^ ^48^ or when used for store and forward teledermatology.^49^ ^50^ However, healthcare professionals who work in primary and secondary care need to be aware of the limitations of algorithm based apps to reliably identify melanomas, and should inform potential smartphone app users about these limitations.

**What is already known on this topic**

Skin cancer is one of the most common cancers in the world, and the incidence is increasing
Algorithm based smartphone applications (“apps”) provide the user with an instant assessment of skin cancer risk and offer the potential for earlier detection and treatment, which could improve survival
A Cochrane review of only two studies that tried to validate algorithm based skin apps suggested that there is a high chance of skin cancers being missed

**What this study adds**

This review identified nine eligible studies that evaluated apps for risk stratification of skin lesions, and showed variable and unreliable test accuracy for six different apps
Studies evaluated apps in selected groups of lesions, using images taken by experts rather than by app users, and many did not identify whether low risk lesions were truly benign
In a rapidly advancing field, quality of evidence is poor to support the use of these apps to assess skin cancer risk in adults with concerns about new or changing skin lesions

## Web extra

Extra material supplied by authors

**Web appendix 1:** Supplementary appendices

**Web appendix 2:** Supplementary figures

## Notes

Contributors: KF and JD contributed equally to this work. KF, JD, and NC undertook the review. All authors contributed to the conception of the work and interpretation of the findings. KF, JD, and JJD drafted the manuscript. All authors critically revised the manuscript and approved the final version. JJD acts as guarantor. The corresponding author attests that all listed authors meet authorship criteria and that no others meeting the criteria have been omitted.

Ethical approval: Ethical approval not required.

Data sharing: No additional data available

The lead authors and manuscript’s guarantor affirm that the manuscript is an honest, accurate and transparent account of the study being reported; that no important aspects of the study have been omitted; and that any discrepancies from the study as planned have been explained.

Dissemination to participants and related patient and public communities: No study participants were involved in the preparation of this systematic review. The results of the review will be summarised in media press releases from the Universities of Birmingham and Nottingham and presented at relevant conferences (including the Centre of Evidence-Based Dermatology 2020 Evidence Based Update Meeting on Keratinocyte carcinomas).
