# Supplementary material for: Algorithm based smartphone apps to assess risk of skin cancer in adults: systematic review of diagnostic accuracy studies
Source: BMJ. 2020 Feb 10;368:m127. doi: 10.1136/bmj.m127 (PMC7190019; doi:10.1136/bmj.m127)
Supplement: Supplementary file 1 — Web appendix 1: Supplementary appendices [file frek052868.ww1.pdf]

Online supplementary appendices

Appendix 1 Search strategies

Database: Ovid MEDLINE(R) 1946 to March Week 5 2019

Search Strategy:

1 melanoma.ti,ab.

2 skin cancer.ti,ab.

3 (skin adj2 (cancer$1 or carcinoma$1 or mass or masses or tumour$1 or tumor$1 or neoplasm$1 or adenoma$1 or epithelioma$1 or lesion$1 or malignan$ or nodule$1)).ti,ab.

4 (pigmented adj2 (lesion$1 or mole$ or nevus or nevi or naevus or naevi or skin)).ti,ab.

5 (melanom$1 or nonmelanoma$1 or non-melanoma$1 or melanocyt$ or non-melanocyt$ or nonmelanocyt$1).ti,ab.

6 or/1-5

7 ((Mobile or cell or cellular or smart) adj phone$1 adj2 app$1 adj2 (app$1 or application$1)).ti,ab.

8 (((smart adj2 device*) or smart) adj handheld).ti,ab.

9 ((mobile or smart) adj telederm$).ti,ab.

10 smartphone$.ti,ab.

11 (DermoScan or SkinVision or Dermlink or SpotCheck).ti,ab.

12 Mole Detective.ti,ab.

13 Spot Check.ti,ab.

14 mHealth.ti,ab.

15 or/7 – 14

16 6 and 15

17 (201607$ or 201608$ or 201609$ or 201610$ or 201611$) or 201612$.ed.

18 16 and 17

19 limit 16 to yr="2016 - 2019"

20 18 or 19

Database: Ovid MEDLINE (Ovid) In-Process & Other Non-Indexed Citations 1946 to April 09, 2019

Search Strategy:

1 melanoma.ti,ab.

2 skin cancer.ti,ab.

3 (skin adj2 (cancer$1 or carcinoma$1 or mass or masses or tumour$1 or tumor$1 or neoplasm$1 or adenoma$1 or epithelioma$1 or lesion$1 or malignan$ or nodule$1)).ti,ab.

4 (pigmented adj2 (lesion$1 or mole$ or nevus or nevi or naevus or naevi or skin)).ti,ab.

5 (melanom$1 or nonmelanoma$1 or non-melanoma$1 or melanocyt$ or non-melanocyt$ or nonmelanocyt$1).ti,ab.

6 or/1-5

7 ((Mobile or cell or cellular or smart) adj phone$1 adj2 app$1 N2 (app$1 or application$1)).ti,ab.

8 (((smart adj2 device*) or smart) adj handheld).ti,ab.

9 ((mobile or smart) adj telederm$).ti,ab.

10 smartphone$.ti,ab.

11 (DermoScan or SkinVision or Dermlink or SpotCheck).ti,ab.

12 Mole Detective.ti,ab.

13 Spot Check.ti,ab.

14 mHealth.ti,ab.

15 or/7 – 14

16 6 and 15

17 (201607$ or 201608$ or 201609$ or 201610$ or 201611$ or 201612$).ed.

18 16 and 17

19 limit 16 to yr="2016 - 2019"

20 18 or 19

Database: Embase (Ovid) 1974 to 2019 April 09

Search Strategy:

1 *melanoma/

2 *skin cancer/

3 (skin adj2 (cancer$1 or carcinoma$1 or mass or masses or tumour$1 or tumor$1 or neoplasm$1 or adenoma$1 or epithelioma$1 or lesion$1 or malignan$ or nodule$1)).ti,ab.

4 (pigmented adj2 (lesion$1 or mole$ or nevus or nevi or naevus or naevi or skin)).ti,ab.

5 (melanom$1 or nonmelanoma$1 or non-melanoma$1 or melanocyt$ or non-melanocyt$ or nonmelanocyt$1).ti,ab.

6 or/1-5

7 ((Mobile or cell or cellular or smart) adj phone$1 adj2 app$1 adj2 (app$1 or application$1)).ti,ab.

8 (((smart adj2 device*) or smart) adj handheld).ti,ab.

9 ((mobile or smart) adj telederm$).ti,ab.

10 smartphone$.ti,ab.

11 (DermoScan or SkinVision or Dermlink or SpotCheck).ti,ab.

12 Mole Detective.ti,ab.

13 Spot Check.ti,ab.

14 mHealth.ti,ab.

15 or/7-14

16 6 and 15

17 limit 16 to yr=2016-2019

18 (201607$ or 201608$ or 201609$ or 201610$ or 201611$ or 201612$).ed.

19 16 and 18

20 17 or 19

21 limit 20 to exclude medline journals

22/02/2019 14:34:58

Cochrane Library (Wiley) 2019 Issue 4

Search strategy

#1 melanoma* or nonmelanoma* or non-melanoma* or melanocyt* or non-melanocyt* or nonmelanocyt* or keratinocyte*

#2 MeSH descriptor: [Melanoma] explode all trees

#3 "skin cancer*"

#4 MeSH descriptor: [Skin Neoplasms] explode all trees

#5 skin near/2 (cancer* or carcinoma* or mass or masses or tumour* or tumor* or neoplasm* or adenoma* or epithelioma* or lesion* or malignan* or nodule*)

#6 nmsc

#7 pigmented near/2 (lesion* or nevus or mole* or naevi or naevus or nevi or skin)

#8 #1 or #2 or #3 or #4 or #5 or #6 or #7

#9 mobile* or smart near/2 phone*

#10 cell next phone*

#11 smartphone*

#12 mole detective or mole map*

#13 DermoScan or SkinVision or Dermalive"

#15 "Spot Check"

#16 mhealth

#17 #9 or #10 or #11 or #12 or #13 or #14 or #15 or #16

#18 #17 and #8

#19 #18 or #126

#20 #125 or #126 with Publication Year from 2016 to 2019

Database : CINAHL Plus (EBSCO) 1937- 25 July 2019

Search strategy:

S1 (MH "Melanoma") OR (MH "Nevi and Melanomas+")

S2 (MH "Skin Neoplasms+")

S3 (skin) N2 (cancer* or carcinoma* or mass or masses or tumor* or tumour* or neoplasm* or adenoma* or epithelioma* or lesion* or malignan* or nodule*)

S4 (pigmented) N2 (lesion* or mole* or nevus or nevi or naevus or naevi or skin)

S5 melanom* or nonmelanoma* or non-melanoma* or melanocyt* or non-melanocyt* or nonmelanocyt*

S6 S1 OR S2 OR S3 OR S4 OR S5

S7 smartphone* or DermoScan or SkinVision or DermLink or SpotCheck

S8 (mobile or cell or cellular or smart) N2 (phone*) N2 (app or application*)

S9 (mole*) N2 (map*)

S10 mole detective

S11 mHealth

S12 S7 or S8 or S9 or S10 or S11

S13 S12 and S6

S14 Limit S13 to yr = 2016 -2019

Database: Science Citation Index SCI-EXPANDED

Timespan=2016-2019

1 TI=(melanom* or nonmelanom* or non-melanoma* or melanocyt* or non-melanocyt* or nonmelanocyt*)

2 TI=((skin) NEAR/2 (cancer* or carcinoma or mass or masses or tumour* or tumor* or neoplasm* or adenoma* or epithelioma* or lesion* or malignan* or nodule*))

3 TI=((pigmented) near/2 (lesion*)

#4   #3 or #2 or #1

#5   ((Mobile or cell or cellular or smart) N2 app* or application*))

#6   (((smart N2 device*) or smart N2 handheld)

#7   ((mobile or smart) N2 (telederm*) or smartphone* or (DermoScan or SkinVision or Dermlink or SpotCheck) or Mole Detective or Spot Check or mHealth)

#8   (mHealth)

#9   #5 or #6 or #7 or #8

#10   #4 and #9

Timespan=2016-2019

Same strategy used for CPCI EXPANDED

DATABASE: Conference Proceedings

EXPANDED Timespan=2016-2019

ZETOC

Conference Proceedings ZETOC Conference search 29 April 2019

Search #	Hits	Search
8	0	any: naevus AND smartphone
7	1	any: skin lesion and smartphone
6	0	any: skin cancer and smart phone
5	0	any: skin lesion and phone
4	0	any: naevus AND phone
3	1	any: skin cancer AND phone
2	1	any: melanoma and smartphone
1	1	any: melanoma AND phone

Appendix 2 Reasons for exclusion

Study	Exclusion reason	Comment
ACTR 2016	not a primary study	teaching SSE; study terminated
ACTR 2016	not a primary study	SSE; phone plus dermoscope attachment
Alonso 2016	conference abstract	store/forward app; dermoscopic attachmenf
Bae 2016	not a test accuracy study	Early phase study developing smartphone app
Bae 2017	wrong target condition	Estimates skin roughness from mobile phone image
Barcaui 2018	wrong index test - no smartphone; TD store/forward	No 'app'; images sent from smartphone via Whatsapp for dermatologist assessment
Braun 2015	not a test accuracy study	case report
Buechi 2017	not a primary study	SR - includes skin cancer test accuracy studies
Burki 2013	not a primary study	not a primary study
Charalambides 2018a	conference abstract	full text Charalambides 2018b)
Charalambides 2018b	not a primary study	SR
Choi 2018	not a primary study	SR of mHealth Approaches in Managing Skin Cancer
Chuchu 2018	not a primary study	SR
Clark 2018	not a primary study	SR
Dahlen Gyllencreutz 2017	wrong index test - smartphone TD store/forward ; dermoscopic attachment	No 'app'; smartphone used for clinical images for TD assessment alongside dermoscopic images
Diniz 2016	wrong index test - smartphone app; attachment required	Requires dermoscopy attachment; otherwise eligible (reports accuracy for test set of lesions)
Do 2018	wrong index test - smartphone app derivation study	app derivation study; uses cross-validation
Doukas 2112	wrong index test - smartphone app derivation study	app; derivation study
Esteva 2017	wrong index test - no smartphone	No app or smartphone use; CAD study
Farkas 2016	conference abstract	CAD derivation; uses attachment (spectral)
Ferrandiz 2017a	wrong index test - no smartphone; TD store/forward	No 'app'; images acquired using Nikon camera and forwarded to dermatologist
Ferrandiz 2017b	wrong index test - no smartphone; TD store/forward	Same as Ferrandiz 2017a; not smartphone study
Ferrero 2013	wrong target population	only analyses melanomas, no benign lesions
Freeman 2018	wrong index test - smartphone app derivation study; attachment requ	Smartphone confocal microscope development study; requires adapter
Gilmore 2018	wrong index test - no smartphone	CAD derivation study using cross-validation; not conducted using smartphone app
Guido 2018a	conference abstract	full text Guido 2018b
Guido 2018b	wrong index test - smartphone app; TD TBP app	TBP app using artificial skin markings
Hubiche 2016	wrong index test - smartphone; TD store/forward	Patient-acquired smartphone images used during consultation
Jafari 2016	wrong index test - no smartphone; CAD derivation	index test (CAD derivation study)
Jahan-Tigh 2016	wrong index test - smartphone microscope; no app	ex vivo use; no app
Janda 2019	not a primary study	study protocol; study potentially eligible (referral accuracy)

Janda 2014	wrong index test - smartphone; SSE TD store/forward	No app; mobile teledermatoscopy
Jaworek-Korjakowska 2018	wrong index test - smartphone app; attachment required	Requires dermoscopy attachment; otherwise eligible (reports accuracy for test set of lesions); 2016 paper (ref 25) is derivation study for the app
Jeong 2018	not a test accuracy study	No accuracy evaluation; describes a store and forward type application
Karagyris 2012	wrong index test - smartphone app derivation study	smartphone app - derivation study
Keske 2016	conference abstract	SSE store/forward; dermoscopic attachment
Kim 2016	not a test accuracy study	No accuracy evaluation; early phase study describing a multispectral smartphone app (requires phone attachment)
Kostopoulos 2016	not a test accuracy study	No accuracy evaluation; early phase study
Kukutsch 2017	duplicate or related publication	duplicate or related publication (see Nabil 2017)
Lai 2015	wrong index test - smartphone TD store/forward ; dermoscopic attachment	index test (TD store/forward; no app; dermoscopy attachment)
MacKinnon 2016	not a test accuracy study	Early phase study developing an app
Manahan 2015	wrong index test - smartphone; TD store/forward - attachment	Requires dermoscopy attachment; evaluates store/forward app
Marek 2018	not a test accuracy study	App not for diagnosis but for recording TBP images for SSE
Marek 2016	not a primary study	Brief review of available apps for TBP
Markun 2017	wrong index test - smartphone; TD store/forward	No 'app'; smartphone used for clinical images for TD assessment alongside dermoscopic images using dermoscopic attachment
Massone 2007	wrong index test - smartphone app; store/forward app	index test (store/forward app)
Massone 2005	wrong index test - smartphone; TD store/forward	TD store/forward
Munia 2017	wrong index test - no smartphone; CAD derivation	Derivation study; uses cross-validation. No app used
NCT 2018	not a primary study	skin self exam; phone plus dermoscope attachment
NCT 2017	not a primary study	teaching SSE; study terminated
Ngoo 2016	conference abstract	see Nabil 2018
Ngoo 2018	conference abstract	see Nabil 2018
Osei-Tutu 2013	wrong index test - smartphone; TD store/forward	TD store/forward
Ramlakhan 2011	wrong index test - smartphone app derivation study	smartphone app - derivation study
Rat 2018	not a primary study	SR of smartphone apps
Resneck 2016	not a test accuracy study	16 websites/apps evaluated using 6 simulated case studies
Silveira 2019	wrong index test - smartphone app; store/forward app	store/forward
Taylor 2018	conference abstract	evaluates dermoscopic attachment
Taylor 2017	conference abstract	evaluates dermoscopic attachment
Udrea 2014	wrong index test - smartphone app derivation study	app; derivation study

Urwin 2017	conference abstract	conf abstract; evaluates store/forward TD
Varma 2011	not a primary study	not a primary study
Vasefi 2017	conference abstract	conf abstract; derivation study
Von Braunmuhl 2015	duplicate or related publication	see Maier 2015 (included study)
Wadhawan 2011	wrong index test - smartphone app derivation study	smartphone app - derivation study
Webster 2017	not a test accuracy study	Uses app to gather observational data about users' moles and other characteristics
Yas 2018	not a primary study	Classifies literature related to smartphone apps
Yu 2011	wrong index test - no smartphone; CAD derivation	index test (CAD derivation study)
Zaidan 2018	not a primary study	Scoping/mapping review; related to Yas 2018
Zink 2017	wrong index test - smartphone; TD store/forward	No 'app'; phone images used for TD
Zouridakis 2015	not a primary study	not a primary study

ACTRN – Australia and New Zealand Clinical Trials Registry ; CAD – computer-assisted diagnosis; SR – systematic review; SSE – skin self examination; TBP – total body photography; TD – teldermatology;

1. ACTR. Efficacy of using mobile teledermoscopy to conduct skin self-examinations in high risk melanoma adults. *Http://wwwwhoint/trialsearch/trial2aspx? Trialid=actrn12616000989448* 2016. https://www.cochranelibrary.com/central/doi/10.1002/central/CN-01877347/full.
2. ACTR. A research study of digitally supported skin self-examination compared to usual care in people treated for localised melanoma. *Http://wwwwhoint/trialsearch/trial2aspx? Trialid=actrn12616001716459* 2016. https://www.cochranelibrary.com/central/doi/10.1002/central/CN-01847856/full.
3. Alonso C, Salerni G, Fernandez Bussy R. Mobile teledermoscopy. A useful tool to evaluate suspicious lesions. Pilot project in Rosario, Argentina. *Melanoma Research* 2016;26 (Supplement 1):e29-e30.
4. Bae JS, Jeon JH, Lee JY, et al. Skin Condition Estimation Using Mobile Handheld Camera. *Etri Journal* 2016;38(4):776-86. doi: 10.4218/etrij.16.0115.0942
5. Bae JS, Lee SH, Choi KS, et al. Robust skin-roughness estimation based on co-occurrence matrix. *Journal of Visual Communication and Image Representation* 2017;46:13-22. doi: 10.1016/j.jvcir.2017.03.003
6. Barcaui CB, Lima PMO. Application of Teledermoscopy in the Diagnosis of Pigmented Lesions. *International Journal of Telemedicine & Applications* 2018;2018:1624073.
7. Braun RP, Marghoob A. High-dynamic-range dermoscopy imaging and diagnosis of hypopigmented skin cancers. *JAMA Dermatology* 2015;151(4):(2p). doi: 10.1001/jamadermatol.2014.4714
8. Buechi R, Faes L, Bachmann LM, et al. Evidence assessing the diagnostic performance of medical smartphone apps: a systematic review and exploratory meta-analysis. *BMJ Open* 2017;7(12):e018280.
9. Burki TK. Diagnostic accuracy of smartphone applications. *Lancet Oncology* 2013;14(3):e90.
10. Charalambides M, Singh S. Use of dermatology apps: A cause for concern or the future of healthcare provision? *British Journal of Dermatology* 2018;179 (Supplement 1):186.
11. Charalambides M, Singh S. Use of dermatology apps: A cause for concern or the future of healthcare provision? *Journal of the National Student Association of Medical Research* 2018;1(2):45-49.
12. Choi J, Cho Y, Woo H. mHealth Approaches in Managing Skin Cancer: Systematic Review of Evidence-Based Research Using Integrative Mapping. *JMIR MHealth and UHealth* 2018;6(8):e164.
13. Chuchu N, Takwoingi Y, Dinnes J, et al. Smartphone applications for triaging adults with skin lesions that are suspicious for melanoma. *Cochrane Database of Systematic Reviews* 2018(12) doi: 10.1002/14651858.cd013192
14. Clark AK, Bosanac S, Ho BR, et al. Systematic review of mobile phone-based teledermatology. *Archives of Dermatological Research* 2018;310(9):675-89. doi: 10.1007/s00403-018-1862-4
15. Dahlen Gyllencreutz J, Paoli J, Bjellerup M, et al. Diagnostic agreement and interobserver concordance with teledermoscopy referrals. *Journal of the European Academy of Dermatology & Venereology* 2017;31(5):898-903.
16. Diniz LE, Ennser K. Melanoma detection using a mobile phone app [9699-30]. *Proceedings of SPIE, the International Society for Optical Engineering* 2016;9699:9699 0V.
17. Do TT, Hoang T, Pomponiu V, et al. Accessible Melanoma Detection Using Smartphones and Mobile Image Analysis. *Ieee Transactions on Multimedia* 2018;20(10):2849-64. doi: 10.1109/tmm.2018.2814346
18. Doukas C, Stagkopoulos P, Kiranoudis CT, et al. Automated skin lesion assessment using mobile technologies and cloud platforms. *Conference Proceedings: Annual International Conference of the IEEE Engineering in Medicine & Biology Society* 2012;2012:2444-7.
19. Esteva A, Kuprel B, Novoa RA, et al. Dermatologist-level classification of skin cancer with deep neural networks. *Nature* 2017;542(7639):115-18.
20. Farkas DL, Vasefi F, MacKinnon NB, et al. Future of care for patients at high risk for melanoma: From multimode, hyperspectral dermoscopy to self-imaging with smartphone. *Melanoma Research* 2016;26 (Supplement 1):e31.
21. Ferrándiz L, Ojeda-Vila T, Corrales A, et al. Internet-based skin cancer screening using clinical images alone or in conjunc tion with dermoscopic images: A randomized teledermoscopy trial. *Journal of the American Academy of Dermatology* 2017;76(4):(7p). doi: 10.1016/j.jaad.2016.10.041
22. Ferrándiz L, Ojeda-Vila T, Corrales A, et al. Impact of dermoscopy on an internet-based skin cancer triage system: Interim results of a randomized study. *Journal of the American Academy of Dermatology* 2017;76(2):(2p). doi: 10.1016/j.jaad.2016.02.1165
23. Ferrero NA, Morrell DS, Burkhart CN. Skin scan: A demonstration of the need for FDA regulation of medical apps on iPhone. *Journal of the American Academy of Dermatology* 2013;68(3):515-16. doi: https://doi.org/10.1016/j.jaad.2012.10.045
24. Freeman EE, Semeere A, Osman H, et al. Smartphone confocal microscopy for imaging cellular structures in human skin in vivo. *Biomedical Optics Express* 2018;9(4):1906-15. doi: 10.1364/boe.9.001906
25. Gilmore SJ. Automated decision support in melanocytic lesion management. *Plos One* 2018;13(9) doi: 10.1371/journal.pone.0203459

26. Guido N, Hagstrom E, Ibler E, et al. A novel total body digital photography smartphone application to detect and monitor skin lesions: A feasibility study. *Journal of the American Academy of Dermatology* 2017;76 (6 Supplement 1):AB19.
27. Guido N, Hagstrom E, Ibler E, et al. A novel total body digital photography smartphone application designed to detect and monitor skin lesions: A pilot study. *J Surg Dermatol* 2018;3(2):177. doi: http://dx.doi.org/10.18282/jsd.v3.i2.177
28. Hubiche T, Valerio L, Boralevi F, et al. Visualization of Patients' Skin Lesions on Their Smartphones: A New Step During Dermatology Visits. *JAMA Dermatology* 2016;152(1):95-7.
29. Jafari MH, Samavi S, Karimi N, et al. Automatic detection of melanoma using broad extraction of features from digital images. *Conference Proceedings: Annual International Conference of the IEEE Engineering in Medicine & Biology Society* 2016;2016:1357-60.
30. Jahan-Tigh RR, Chinn GM, Rapini RP. A Comparative Study Between Smartphone-Based Microscopy and Conventional Light Microscopy in 1021 Dermatopathology Specimens. *Archives of Pathology & Laboratory Medicine* 2016;140(1):86-90.
31. Janda M, Horsham C, Koh U, et al. Redesigning Skin Cancer Early Detection and Care Using a New Mobile Health Application: Protocol of the SKIN Research Project, a Randomised Controlled Trial. *Dermatology* 2019;235(1):11-18.
32. Janda M, Loescher LJ, Banan P, et al. Lesion selection by melanoma high-risk consumers during skin self-examination using mobile teledermoscopy. *JAMA dermatology* 2014; 150(6). https://www.cochranelibrary.com/central/doi/10.1002/central/CN-00995267/full
https://jamanetwork.com/journals/jamadermatology/articlepdf/1829636/dld130031.pdf.
33. Jaworek-Korjakowska J, Kleczek P. eSkin: Study on the Smartphone Application for Early Detection of Malignant Melanoma. *Wireless Communications & Mobile Computing* 2018 doi: 10.1155/2018/5767360
34. Jeong CW, Joo SC. Skin care management support system based on cloud computing. *Multimedia Tools and Applications* 2018;77(8):9885-96. doi: 10.1007/s11042-017-5521-0
35. Karargyris A, Karargyris O, Pantelopoulos A, et al. DERMA/care: An advanced image-processing mobile application for monitoring skin cancer. *2012 Ieee 24th International Conference on Tools with Artificial Intelligence (Ictai 2012), Vol 2* 2012:1-7. doi: 10.1109/ictai.2012.180
36. Keske R, Armstrong GT, Davine J, et al. Advancing Survivors' Knowledge (ASK) about skin cancer study: A randomized intervention within the Childhood Cancer Survivor Study (CCSS). *Journal of Clinical Oncology Conference* 2016;34(3 SUPPL. 1)
37. Kim S, Cho D, Kim J, et al. Smartphone-based multispectral imaging: system development and potential for mobile skin diagnosis. *Biomedical Optics Express* 2016;7(12):5294-307.
38. Kostopoulos S, Glotsos D, Asvestas P, et al. AN ENSEMBLE TEMPLATE MATCHING AND CONTENT-BASED IMAGE RETRIEVAL SCHEME TOWARDS EARLY STAGE DETECTION OF MELANOMA. *Image Analysis & Stereology* 2016;35(3):137-48. doi: 10.5566/ias.1446
39. Kukutsch NA. Skin cancer apps: Possibilities and impossibilities. *Nederlands Tijdschrift voor Dermatologie en Venereologie* 2017;27(4):155-57.
40. Lai I, Ko J, Pathipati A. DermLens: Device for mobile teledermatology. *Journal of the American Academy of Dermatology* 2015;1):AB88.
41. MacKinnon N, Vasefi F, Booth N, et al. Melanoma detection using smartphone and multimode hyperspectral imaging [9711-29]. *Proceedings of SPIE, the International Society for Optical Engineering* 2016;9711:9711 17.
42. Manahan MN, Soyer HP, Loescher LJ, et al. A pilot trial of mobile, patient-performed teledermoscopy. *British journal of dermatology* 2015; 172(4). https://www.cochranelibrary.com/central/doi/10.1002/central/CN-01068310/full
https://onlinelibrary.wiley.com/doi/pdf/10.1111/bjd.13550.
43. Marek AJ, Chu EY, Ming ME, et al. Impact of a smartphone application on skin self-examination rates in patients who are new to total body photography: a randomized controlled trial. *Journal of the american academy of dermatology* 2018; 79(3). https://www.cochranelibrary.com/central/doi/10.1002/central/CN-01629021/full
https://www.sciencedirect.com/science/article/pii/S0190962218302111?via%3Dihub.
44. Marek AJ, Chu EY, Ming ME, et al. Assessment of smartphone applications for total body digital photography-guided skin exams by patients. *Journal of the American Academy of Dermatology* 2016;75(5):1063-64. doi: 10.1016/j.jaad.2016.06.005
45. Markun S, Scherz N, Rosemann T, et al. Mobile teledermatology for skin cancer screening: A diagnostic accuracy study. *Medicine* 2017;96(10):e6278.
46. Massone C, al e. Cellular phones in clinical dermatology. *JAMA Dermatology* 2005;141(10):1319-20. doi:

10.1001/archderm.141.10.1319
47. Massone C, Hofmann-Wellenhof R, Ahlgrimm-Siess V, et al. Melanoma screening with cellular phones. *PLoS ONE [Electronic Resource]* 2007;2(5):e483.
48. Munia TTK, Alam MN, Neubert J, et al. Automatic diagnosis of melanoma using linear and nonlinear features from digital image. *Conference Proceedings: Annual International Conference of the IEEE Engineering in Medicine & Biology Society* 2017;2017:4281-84.
49. Nct. Teaching Skin Self-Examination to First-degree Relatives of Melanoma Patients Using Mobile App Technology. *Https://clinicaltrialsgov/show/nct03177941* 2017. https://www.cochranelibrary.com/central/doi/10.1002/central/CN-01594729/full.
50. Nct. A Research Study of Patient-led Surveillance Compared to Clinician-led Surveillance in People Treated for Localised Melanoma. *Https://clinicaltrialsgov/show/nct03581188* 2018. https://www.cochranelibrary.com/central/doi/10.1002/central/CN-01660955/full.
51. Ngoo A, Finnane A, McMeniman E, et al. Efficacy of smartphone applications in high-risk pigmented lesions. *Australasian Journal of Dermatology* 2018;59(Supplement 1):88.
52. Ngoo A, Finnane A, Tan JM, et al. Efficacy of smartphone applications in detecting melanoma. *Australasian Journal of Dermatology* 2016;57 (Supplement 2):34.
53. Osei-Tutu A, al e. Mobile teledermatology in Ghana: sending and answering consults via mobile platform. *J Am Acad Dermatol* 2013;69:e90-e91.
54. Ramlakhan K, Shang Y, Ieee. A Mobile Automated Skin Lesion Classification System. 2011 23rd Ieee International Conference on Tools with Artificial Intelligence, 2011:138-41.
55. Rat C, Hild S, Rault Serandour J, et al. Use of Smartphones for Early Detection of Melanoma: Systematic Review. *Journal of Medical Internet Research* 2018;20(4):e135.
56. Resneck JS, Abrouk M, Steuer M, et al. Choice, Transparency, Coordination, and Quality Among Direct-to-Consumer Telemedicine Websites and Apps Treating Skin Disease. *Jama Dermatology* 2016;152(7):768-75. doi: 10.1001/jamadermatol.2016.1774
57. Silveira CEG, Carcano C, Mauad EC, et al. Cell phone usefulness to improve the skin cancer screening: preliminary results and critical analysis of mobile app development. *Rural and Remote Health* 2019;19(1) doi: 10.22605/rrh4895
58. Taylor J, Oakley A. Is a dermatoscope intended for consumer use adequate to diagnose skin cancer? *Australasian Journal of Dermatology* 2017;58 (4):338-39.
59. Taylor J, Wang M, Oakley A. Is a dermatoscope intended for consumer use adequate to diagnose melanoma? *Australasian Journal of Dermatology* 2018;59 (Supplement 1):117.
60. Real-time acquisition of quality verified nonstandardized color images for skin lesions risk assessment — A preliminary study. 2014 18th International Conference on System Theory, Control and Computing (ICSTCC); 2014 17-19 Oct. 2014.
61. Urwin R, Bhutani R, Guest C, et al. An Apple (or Android) a day keeps the 2-week-wait targets at bay. *British Journal of Dermatology* 2017;177 (Supplement 1):187.
62. Varma S. Mobile teledermatology for skin tumour screening. *British Journal of Dermatology* 2011;164(5):939-40.
63. Vasefi F, MacKinnon N, Booth N, et al. Melanoma surveillance by multimode, hyperspectral dermoscopy and self-imaging using smartphone in high-risk patients. *Journal of the American Academy of Dermatology* 2017;76 (6 Supplement 1):AB168.
64. Von Braunmühl T. Smartphone apps for skin cancer diagnosis? The munich studie. *Kosmetische Medizin* 2015;36, NUMB 4:152-57.
65. Wadhawan T, Situ N, Rui H, et al. Implementation of the 7-point checklist for melanoma detection on smart handheld devices. *Conference Proceedings: Annual International Conference of the IEEE Engineering in Medicine & Biology Society* 2011;2011:3180-3.
66. Webster DE, Suver C, Doerr M, et al. Data Descriptor: The Mole Mapper Study, mobile phone skin imaging and melanoma risk data collected using ResearchKit. *Scientific Data* 2017;4 doi: 10.1038/sdata.2017.5
67. Yas QM, Zaidan AA, Zaidan BB, et al. A Systematic Review on Smartphone Skin Cancer Apps: Coherent Taxonomy, Motivations, Open Challenges and Recommendations, and New Research Direction. *Journal of Circuits Systems and Computers* 2018;27(5) doi: 10.1142/s0218126618300039
68. Yu LS, Joseph A, Lindsley EH, et al. Polarization-Sensitive Digital Dermoscopy For Image Processing-Assisted Evaluation Of Atypical Nevi: Towards Step-Wise Detection Of Melanoma. *Imaging, Manipulation, and Analysis of Biomolecules, Cells, and Tissues Ix* 2011;7902 doi: 10.1117/12.891083
69. Zaidan AA, Zaidan BB, Albahri OS, et al. A review on smartphone skin cancer diagnosis apps in evaluation and benchmarking: coherent taxonomy, open issues and recommendation pathway solution. *Health and Technology* 2018;8(4):223-38.

70. Zink A, Kolbinger A, Leibl M, et al. [Teledermoscopy by mobile phones : Reliable help in the diagnosis of skin lesions?]. *Hautarzt* 2017;68(11):890-95.
71. Zouridakis G, Wadhawan T, Situ N, et al. Melanoma and other skin lesion detection using smart handheld devices. *Methods in Molecular Biology* 2015;1256:459-96.

Appendix 3 QUADAS-2

Item	Response (delete as required)
**PARTICIPANT SELECTION (1) - RISK OF BIAS**	
1) Was a consecutive or random sample of participants or images enrolled?	**Yes -** if paper states consecutive or random, or if *all* participants meeting explicit study eligibility criteria within a specified time frame were clearly included<br>**No** – if paper describes other method of sampling<br>**Unclear** – if participant sampling not described
2) Was a case-control design avoided?	**Yes -** if case-control design clearly not used<br>**No** – if study described as case-control or describes sampling specific numbers of participants with particular diagnoses<br>**Unclear** – if not clearly described or you have any concerns that the authors have not selected a series of participants
3) Did the study avoid inappropriate exclusions, e.g.<br>• 'difficult to diagnose' lesions not excluded<br>• lesions not excluded on basis of disagreement between evaluators or histopathologists	**Yes -** if inappropriate exclusions were avoided<br>**No** – if lesions were excluded that might affect test accuracy, e.g. 'difficult to diagnose' lesions, OR where disagreement between evaluators was observed<br>**Unclear** – if not clearly reported but there is suspicion that difficult to diagnose lesions may have been excluded
**Could the selection of participants have introduced bias?**<br>*If answers to all of questions 1) AND 2) AND 3) 'Yes' :*<br>*If answers to any one of questions 1) OR 2) OR 3) 'No' :*<br>*If answers to any one of questions 1) OR 2) OR 3) 'Unclear':*	 ***Risk is Low***<br>***Risk is High***<br>***Risk Unclear***
**PARTICIPANT SELECTION (1) - CONCERNS REGARDING APPLICABILITY**	
1) Are the included patients and chosen study setting appropriate to answer the review question, i.e. are the study results generalisable?<br><br>This item is not asking whether exclusion of certain patient groups might bias the study's results (as in Risk of Bias above), but is asking whether the chosen study participants and setting are appropriate to answer our review question. Because we are looking to establish test accuracy in both primary presentation and referred participants, a study could be appropriate for one setting and not for the other, or it	**Yes** – if patients included in the study appear to be generally representative of those who might present in a usual practice setting<br>**No** if study participants were restricted to those in lesion subgroups, e.g. melanocytic only, or small lesions only, if only excised lesions were included, or lesions were selected from referred populations rather than selected by GPs in a primary care setting<br>**Unclear –** if insufficient details are provided to determine the generalisability of study participants

Item	Response (delete as required)
could be unclear as to whether the study can appropriately answer either question.	
2) Did the study **avoid including** participants with multiple lesions?	***Yes*** – if the difference between the number of included lesions and number of included participants is less than 5%<br>**No** – if the difference between the number of included lesions and number of included participants is greater than 5%<br>**Unclear –** if it is not possible to assess
Is there concern that the included participants do not match the review question?<br>*If the answer to question 1) and 2) 'Yes':*<br>*If the answer to question 1) or 2) 'No':*<br>*If the answer to question 1) or 2) 'Unclear':*	 <br>***Concern is Low***<br>***Concern is High***<br>***Concern is Unclear***
**INDEX TEST (2) - RISK OF BIAS (to be completed per test evaluated)**	
1) Was the index test or testing strategy result interpreted without knowledge of the results of the reference standard?	**Yes -** if index test described as interpreted without knowledge of the reference standard result or, for prospective studies, if index test is always conducted and interpreted prior to the reference standard<br>**No** – if index test described as interpreted in knowledge of reference standard result<br>**Unclear** – if index test blinding is not described
2) Was the diagnostic threshold at which the test was considered positive (i.e. melanoma, BCC or cSCC present) pre-specified?	**Yes -** if threshold was pre-specified (i.e. prior to analysing study results), ie results were not data driven<br>**No -** if threshold was not pre-specified but was selected after analysis of results usually to maximise sensitivity and/or specificity, or multiple thresholds were tested<br>**Unclear -** if not possible to tell whether or not diagnostic threshold was pre-specified
Could the conduct or interpretation of the index test have introduced bias?<br>❖ FOR NC STUDIES<br>*If answers to questions 1) and 2) 'Yes'*<br>*If answers to either questions 1) or 2) 'No'*<br>*If answers to either questions 1) or 2) 'Unclear' :*	 <br><br>***Risk is Low***<br>***Risk is High***<br>***Risk is Unclear***
**INDEX TEST (2) - CONCERN ABOUT APPLICABILITY**	
1) Was the test applied and interpreted in a clinically applicable manner?	**Yes** – in-person evaluation and single observer result presented<br>**No -** either image based and/or average or consensus result presented

Item	Response (delete as required)
	**Unclear** – if can't tell
2) Were thresholds or criteria for diagnosis reported in sufficient detail to allow replication?<br><br>Study results can only be reproduced if the diagnostic threshold is described in sufficient detail. This item applies equally to studies using pattern recognition and those using checklists or algorithms to aid test interpretation	**Yes** – If the criteria for diagnosis of the target disorder were reported in sufficient detail to allow replication. If the study does not describe the threshold in detail BUT evaluates an established test/algorithm AND provides a citation to a previous study of the test in the Methods or Results, then respond Yes.<br>**No** – if the criteria for diagnosis of the target disorder were not reported in sufficient detail to allow replication<br>**Unclear** – If some but not sufficient information on criteria for diagnosis to allow replication were provided. If the study does not describe the threshold in detail BUT evaluates an established test/algorithm but with NO citation to a previous study of the test in the methods, then respond Unclear.
Is there concern that the index test, its conduct, or interpretation differ from the review question?<br>*If answers to questions 1) and 2)) 'Yes'*<br>*If answers to questions 1) or 2)) 'No'*<br>*If answers to questions 1) OR 2) 'Unclear'*	 <br>***Concern is Low***<br>***Concern is High***<br>***Concern is Unclear***
**REFERENCE STANDARD (3) - RISK OF BIAS**	
1) Is the reference standard likely to correctly classify the target condition?	**Yes** – if all disease positive participants and >=80% of disease negative participants had histological confirmation of final disease status<br>**No** – If a final diagnosis for any disease positive participant or for >20% of disease negative participants was reached without histopathology<br>**Unclear** – if the method of final diagnosis was not reported

Item	Response (delete as required)
2) Were the reference standard results interpreted without knowledge of the results of the index test?	**For studies comparing smartphone apps against a histological reference standard**<br>**Yes** – if the histological reference standard diagnosis was reached blinded to the index test result<br>**No** – if the histological reference standard diagnosis was reached with knowledge of the index test result<br>**Unclear** – if blinded reference test interpretation was not clearly reported<br><br>If the histopathologist is described as 'blinded' with no further detail as to whether the blinding applies to both index test or to clinical information (prior testing), we will assume that blinding is to the index test result only, unless further detail is provided<br><br>**For studies comparing smartphone apps against a face-to-face expert diagnosis**<br>**Yes -** if the face-to-face reference standard diagnosis was described as interpreted without knowledge of the teledermatology diagnosis (e.g. the remote and face to face diagnosis was made by two different dermatologists)<br>**No -** if the face-to-face reference standard diagnosis was made with knowledge of the teledermatology diagnosis or was made by the same dermatologist within a month of the remote image-based diagnosis<br>**Unclear** – if it is not possible to tell whether knowledge of the teledermatology diagnosis could have influenced the reference standard diagnosis
Could the reference standard, its conduct, or its interpretation have introduced bias?<br>*If answers to questions 1) AND 2 'Yes'*<br>*If answers to questions 1) OR 2) 'No':*<br>*If answers to questions 1) OR 2) 'Unclear':*	 <br>***Risk is Low***<br>***Risk is High***<br>***Risk is Unclear***
**REFERENCE STANDARD (3) - CONCERN ABOUT APPLICABILITY**	
1) Expert opinion (with no histological confirmation) was not used as a reference standard	**Yes** - if expert opinion was not used as a reference standard for any participant<br>**No** - if expert opinion was used as a reference standard for any participant<br>**Unclear** - if not clearly reported
2) Was the reference standard diagnosis ascertained by an experienced histopathologist or by a dermatopathologist, or by an experienced observer (face-to-face diagnosis)?	**Yes –** if final diagnosis was reported to be ascertained by an experienced observer

Item	Response (delete as required)
	**No –** if final diagnosis was reported to be ascertained by a less experienced observer<br>**Unclear** – if the experience/qualifications of the observer was not reported
Is there concern that the target condition as defined by the reference standard does not match the review question?<br>If answer to either questions 1) or 2), 'Yes':<br>If answer to either questions 1) OR 2), 'No':<br>3) If answer to either questions 1) OR 2), 'Unclear':	***Concern is Low***<br>***Concern is High***<br>***Concern is Unclear***
**FLOW AND TIMING (4): RISK OF BIAS**	
1) Was there an appropriate interval between index test and reference standard?<br><br>*a)* For histopathological reference standard, was the interval between index test and reference standard <= 1month?<br><br>*b)* If the reference standard includes clinical follow-up of borderline/benign appearing lesions, was there a minimum follow-up following application of index test(s) of at least:<br>• 3 months for melanoma or cSCC<br>• 6 months for BCC?	**Yes** – if study reports <=1 month between index and reference standard<br>**No** – if study reports >1 month between index and reference standard<br>**Unclear** – if study does not report interval between index and reference standard<br><br>**Yes** – if study reports >=3 (or 6) months follow-up<br>**No** – if study reports <3 (or 6) months follow-up<br>**Unclear** – if study does not report length of clinical follow-up
2) Did all participants receive the same reference standard?	**Yes** – if all participants underwent the same reference standard<br>**No** – if more than one reference standard was used<br>**Unclear** – if not clearly reported
3) Were all participants included in the analysis?	**Yes** – if all participants were included in the analysis

Item	Response (delete as required)
	**No** – if some participants were excluded from the analysis<br>**Unclear**– if not clearly reported
Could the participant flow have introduced bias?<br>❖ **FOR NON COMPARATIVE and BPC STUDIES**<br>*-If answers to questions 1)AND 2) AND 3) : 'Yes':*<br>*-If answers to any one of questions 1) OR 2)OR 3) 'No' : If answers to any one of questions 1) OR 2)OR 3) 'Unclear' :*<br>4)	 <br>***Risk is Low***<br>***Risk is High***<br>***Risk is Unclear***
