# Supplementary material for: Algorithm based smartphone apps to assess risk of skin cancer in adults: systematic review of diagnostic accuracy studies
Source: BMJ. 2020 Feb 10;368:m127. doi: 10.1136/bmj.m127 (PMC7190019; doi:10.1136/bmj.m127)
Supplement: Supplementary file 2 — Web appendix 2: Supplementary figures [file frek052868.ww2.pdf]

**Supplementary Figure 1 PRISMA flow diagram of study inclusion**

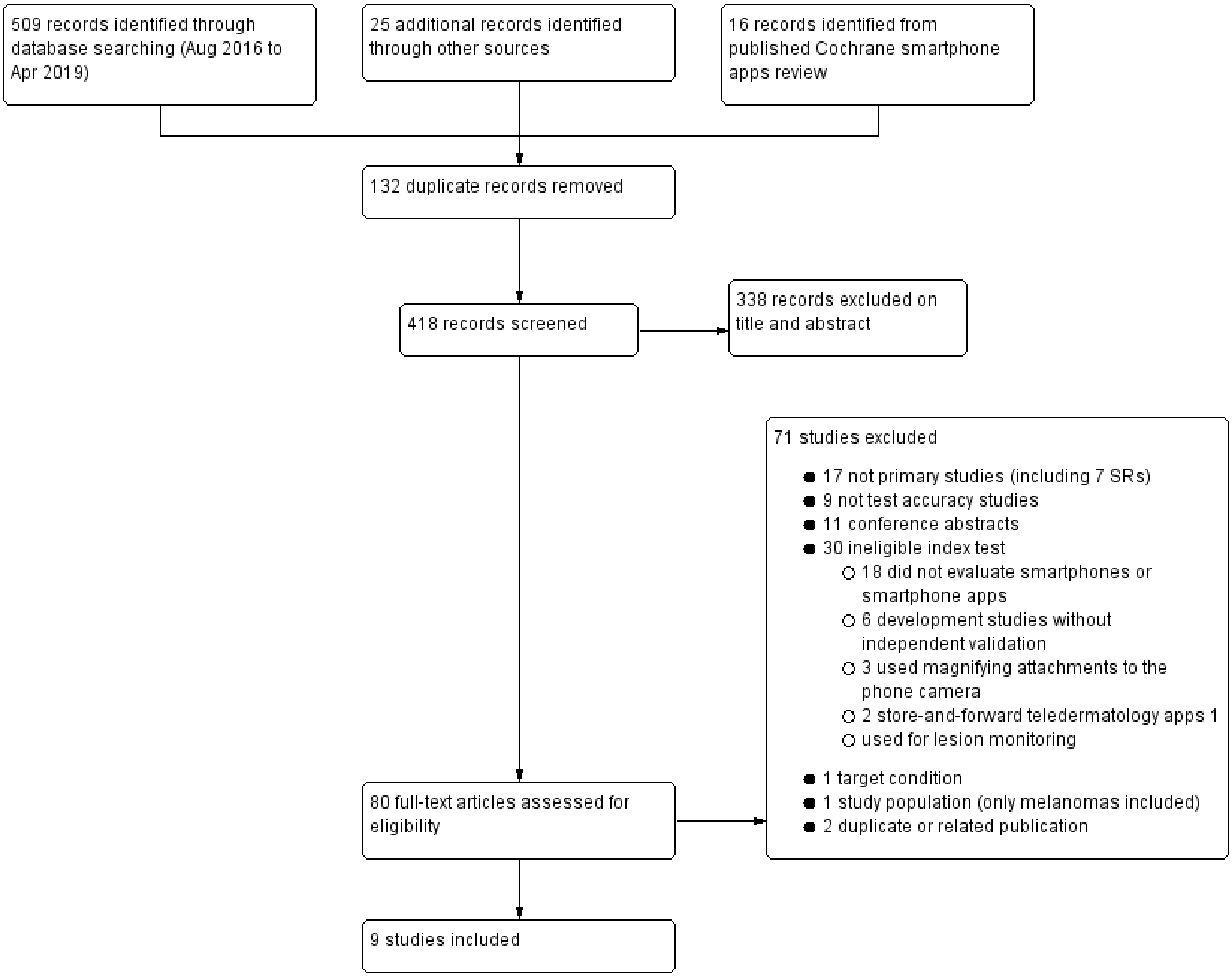

**Supplementary Figure 2 Forest plot of sensitivity and specificity for evaluations of all other identified apps**

Uncertain availability - all thresholds - accuracy compared to histology with or without follow-up

| Study | TP | FP | FN | TN | Mobile platform | Unevaluables | Sensitivity (95% CI) | Specificity (95% CI) |
|---|---|---|---|---|---|---|---|---|
| Chadwick 2014 - Dr Mole - H vs M/L | 4 | 4 | 1 | 6 | Android | Excluded a priori | 0.80 [0.28, 0.99] | 0.60 [0.26, 0.88] |
| Chadwick 2014 - Dr Mole - H/M vs L | 5 | 6 | 0 | 4 | Android | Excluded a priori | 1.00 [0.48, 1.00] | 0.40 [0.12, 0.74] |
| Chadwick 2014 - SpotMole - H vs L | 4 | 4 | 1 | 6 | Android | Excluded a priori | 0.80 [0.28, 0.99] | 0.60 [0.26, 0.88] |

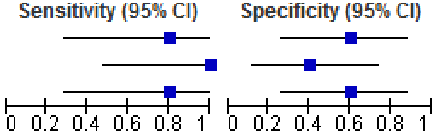


Uncertain availability - all thresholds - accuracy compared to expert recommendation for further investigation/intervention

| Study | TP | FP | FN | TN | Mobile platform | Unevaluables | Sensitivity (95% CI) | Specificity (95% CI) |
|---|---|---|---|---|---|---|---|---|
| Ngoo 2018 - Dr Mole - H vs M/L | 9 | 0 | 33 | 15 | Android | 0 | 0.21 [0.10, 0.37] | 1.00 [0.78, 1.00] |
| Ngoo 2018 - Spotmole (direct) - H vs L | 18 | 3 | 24 | 12 | Android | 0 | 0.43 [0.28, 0.59] | 0.80 [0.52, 0.96] |
| Ngoo 2018 - Spotmole (indirect) - H vs L | 18 | 4 | 24 | 11 | Android | 0 | 0.43 [0.28, 0.59] | 0.73 [0.45, 0.92] |

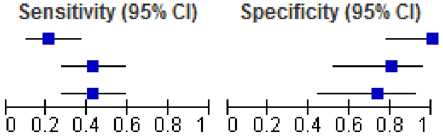


Withdrawn apps - all thresholds - accuracy compared to histology with or without follow-up

| Study | TP | FP | FN | TN | Mobile platform | Unevaluables | Sensitivity (95% CI) | Specificity (95% CI) |
|---|---|---|---|---|---|---|---|---|
| Chadwick 2014 - MelApp - H vs M/L | 0 | 0 | 4 | 5 | Android | 6 | 0.00 [0.00, 0.60] | 1.00 [0.48, 1.00] |
| Chadwick 2014 - MelApp - H/M vs L | 1 | 0 | 3 | 5 | Android | 6 | 0.25 [0.01, 0.81] | 1.00 [0.48, 1.00] |
| Chadwick 2014 - Mole Detective - H vs M/L | 4 | 8 | 1 | 2 | Android | Excluded a priori | 0.80 [0.28, 0.99] | 0.20 [0.03, 0.56] |
| Chadwick 2014 - Mole Detective - H/M vs L | 5 | 8 | 0 | 2 | Android | Excluded a priori | 1.00 [0.48, 1.00] | 0.20 [0.03, 0.56] |
| Robson 2012 - MelApp - H vs M/L | 1 | 2 | 1 | 17 | iOS | 14 | 0.50 [0.01, 0.99] | 0.89 [0.67, 0.99] |
| Robson 2012 - MelApp - H/M vs L | 1 | 4 | 1 | 15 | iOS | 14 | 0.50 [0.01, 0.99] | 0.79 [0.54, 0.94] |

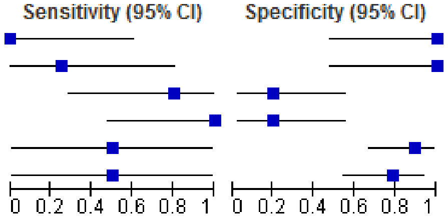


Unidentified apps - all thresholds - accuracy compared to histology with or without follow-up

| Study | TP | FP | FN | TN | Mobile platform | Unevaluables | Sensitivity (95% CI) | Specificity (95% CI) |
|---|---|---|---|---|---|---|---|---|
| Dorairaj 2017 - App A - H vs M/L | 1 | 5 | 8 | 12 | NR | 6 | 0.11 [0.00, 0.48] | 0.71 [0.44, 0.90] |
| Dorairaj 2017 - App A - H/M vs L | 9 | 14 | 0 | 3 | NR | 6 | 1.00 [0.66, 1.00] | 0.18 [0.04, 0.43] |
| Wolf 2013 - App 1 - H vs L | 42 | 74 | 18 | 48 | NR | 6 | 0.70 [0.57, 0.81] | 0.39 [0.31, 0.49] |
| Wolf 2013 - App 2 - H vs L | 40 | 80 | 18 | 47 | NR | 3 | 0.69 [0.55, 0.80] | 0.37 [0.29, 0.46] |
| Wolf 2013 - App 3 - H vs M/L | 4 | 7 | 55 | 104 | NR | 17 | 0.07 [0.02, 0.16] | 0.94 [0.87, 0.97] |
| Wolf 2013 - App 3 - H/M vs L | 32 | 43 | 27 | 68 | NR | 17 | 0.54 [0.41, 0.67] | 0.61 [0.52, 0.70] |

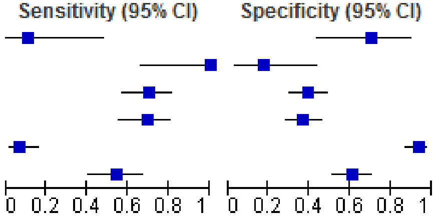


Where possible, data are presented considering only high risk results as test positive, and also considering both high and moderate risk results as test positive.

Spotmole (direct) – uses images taken from within the app; Spotmole (indirect) – uploads images from the smartphone photo library; FN – false negative, FP – false positive, H – high risk, L – low risk, M – moderate risk, TN – true negative, TP – true positive
